# The experiential basis of concepts: integrating embodied and enactive accounts

**DOI:** 10.3389/fpsyg.2025.1710102

**Published:** 2026-01-12

**Authors:** Thomas Fuchs

**Affiliations:** Clinic for General Psychiatry and Department of Philosophy, Heidelberg University, Heidelberg, Germany

**Keywords:** embodied concepts, conceptual metaphors, phenomenology, enactivism, participatory sense-making, neurobiology, language acquisition, cultural evolution

## Abstract

The paper argues for an embodied and enactive view of linguistic concepts as a solution to the “scaling up” problem, namely the transition from embodied experience to symbolic and abstract thought. Drawing on phenomenology, neurobiology, conceptual metaphor theory and enactivism, it aims to demonstrate the constitutive role of the body and intersubjectivity in concept formation. Concrete concepts (“chair”, “table”, etc.) emerge from sensorimotor interactions with the environment which are transformed into simulated actions, while abstract concepts—such as “space”, “time”, “truth”, and others—arise both through metaphorical extensions of bodily experience and participatory sense-making in social contexts. Neurobiological findings support this view, showing strong connections between language processing, sensorimotor and social brain systems, and tracing language evolution to exaptation or reuse of motor coordination areas. Phenomenological analysis then highlights how bodily or operative intentionality underlies grammatical structures, and how concepts retain their roots in action and interaction even when abstracted. As examples, the study explores container schemas as the embodied basis of categorization and analyzes the bodily origins of space, time, causality, and moral concepts. In sum, concepts are not free-floating symbols but remain anchored in corporeal and intersubjective experience, thus integrating embodiment, language, and culture. Human reason proves to be not disembodied, but fundamentally rooted in embodied interaction and intersubjective practice.

The body, in so far as it has ‘behavior patterns', is that strange object which uses its own parts as a general system of symbols for the world, and through which we can consequently ‘be at home in' that world, ‘understand' it and find significance in it.

Merleau-Ponty (1962, p. 212)

## 1 Introduction

Cognitive science has traditionally followed the computational model of mind, according to which the external world is mirrored in the mind as a system of mental representations in the brain. Thinking was then considered a formal manipulation of internal symbols that had no intrinsic relation to the body and its environment. Concepts were regarded as abstract, amodal, and arbitrary, represented in a symbolic “language of thought” (Fodor, 1975, 1987) according to formal syntactic rules. However, concepts of 4e cognition, embodiment, and enactivism challenge this fundamental separation of mind and body. According to them, there is no principled gap between “lower” and “higher” cognitive functions, that is, between our embodied interactions with the environment and the symbolic sphere of language and concepts through which we grasp this environment, categorize it, and communicate with others (Barsalou, 2008, 2012; Di Paolo et al., 2018). From this point of view, human cognition is inseparable from our active engagement in the world. On the neurobiological level, this means that the sensorimotor systems of the brain are crucially involved in language and conceptual thought.

A well-known challenge for such a research program is to explain the “scaling up” (Kiverstein and Rietveld, 2021), that is, the transition from embodied experience to the formation and manipulation of abstract concepts. Such concepts can be used in a virtual mode, namely for thinking, planning, remembering, and communicating with others. Whereas perception and action can be described in terms of agent-environment dynamics, higher cognition is characterized by the ability to deal with absent or merely possible objects, with counterfactual states of affairs, and with general or abstract concepts. Can such forms of cognition be accounted for in terms of embodiment and enaction?

If we assume that reason and thinking developed from the embodied interactions of humans with their environment and with their peers, then there are good reasons to argue that the body should continue to play a constitutive role in the formation, acquisition, and use of both concrete and abstract concepts. This continuity from the cultural evolution of language to its ontogenetic acquisition in early childhood is the central characteristic of the “symbolic species” (Deacon, 1998): every child repeats and reenacts the embodied-interactive development of human language in its early socialization (Tomasello, 1999). If human reason is fundamentally based on language, then there is also an “embodied reason” whose structures do not consist of purely mental categories, but of bodily concepts primarily experienced through action and interaction. This is also particularly relevant in light of recent advances of Large Language Models that simulate our embodied language and give the impression that it is nothing more than a manipulation and rearrangement of disembodied symbols.

In what follows, I will argue for the embodied basis of concepts, making use of the phenomenology of embodiment, of neurobiological findings, and in particular of Lakoff and Johnson's *conceptual metaphor theory* (CMT), which provides a plausible proposal for at least a partial solution to the scaling up problem (Lakoff and Johnson, 1980, 1999). I will start with some conceptual clarifications (1), then move on to the embodied basis of concrete concepts (2), also examining the neurobiological findings which support it (3). Then I turn to embodied metaphors in abstract concepts such as space, time, causality, and certain concepts of morality (4). Furthermore, I present more recent critiques of CMT based on empirical evidence, which show that the theory cannot fully explain abstract concepts. As I argue, it needs to be expanded by an enactive conception of language based on social interaction and participatory meaning-making, which is outlined at the end (5).

## 2 Categories and concepts of experience

Categories are formed in the course of embodied interaction with the environment. They are the cognitive structures that differentiate aspects of our experience and classify them as distinct types. Such categories are not initially tied to linguistic concepts. Babies already categorize in the first months of life, long before language acquisition, distinguishing between living and non-living objects, humans and animals, dogs and cats, or furniture and cars; this can be demonstrated by the differential attention they allocate in habituation tasks (Pauen, 2002; Poulin-Dubois and Pauen, 2017). These primary categories are based on the similarity of the perceived gestalt on the one hand, and on the typical motor interactions with the object on the other (Gallese and Lakoff, 2005). Thus, a chair has a certain shape and it is used for sitting in a certain position.

Language acquisition builds on these pre-conceptual categories (Fuchs, 2016). Linguistic concepts are the mental structures we use to name categories of experience we have already formed, but also to differentiate them further; dogs become sheepdogs, terriers, or mastiffs. Concepts are usually formed around prototypes from which certain degrees of deviation are possible: Tables are flat surfaces that can have four or three legs, but no backrests, for then they turn into chairs; they can also turn into stools, if they are too low. A cat must not bark, otherwise doubts arise as to whether it really is a cat. Nevertheless, the child must understand that the word stands for prototypes of things, i.e., it refers to a conceptual generality with variability.

If concepts are embodied in a strong sense, then bodily processes, whether interoceptive or sensorimotor, are constitutively involved in their formation and use. This seems quite plausible in the case of tables or chairs: without actively interacting with tables (e.g., sitting at them) or chairs (e.g., sitting on them), children would not be able to form their concepts, because they do not yet know or understand definitions, and mere images of tables or chairs do not convey their function which is necessary for the concept. Concepts are thus formed through functional equivalences and affordances of objects: A table is something you can sit on, a spoon something you can use to scoop food into your mouth. A fork is something you can use to spear food, which is difficult to do with a spoon, and so on.

On the other hand, there are of course more abstract concepts. Lakoff and Johnson (1999) therefore distinguish between primary and secondary stages of concept formation:

a Primary, basic-level or concrete concepts are derived from interoceptive bodily experience—e.g. warmth, cold, hunger, thirst—or from sensorimotor interaction with objects such as animals, stones, balls or tables.
b Secondary, higher-level or abstract concepts, on the other hand, are derived from the use of primary concepts as *metaphors*. These “conceptual metaphors” form the basis for our concepts, not of objects, but of *relationships and connections* between ourselves and objects or other persons, and thus for more abstract concepts such as space, time, causality, emotions, or moral concepts.

This second stage is of course particularly significant for the idea of an embodied reason. But let us consider the first step, the direct origin of concepts from embodied, and in particular sensorimotor, experience.

## 3 Basic level concepts

First of all, it is noticeable that we often use our body or body parts to designate objects: We speak of the letterhead, the hand of a clock, the eye of a needle, the tongue of a glacier, or the resonating body of a violin. Obviously, a mimetic component of perception plays an important role here: Our lived body spontaneously assimilates the shape or function of things, so that these are experienced as “quasi-bodies”. The sensorimotor body schema extends and virtually incorporates objects, their shape and motion, as if it were in their place (Fuchs and De Jaegher, 2009).^1^ Through its position and motion in space, the body also provides the basis for all concepts of direction and locality: above, below, behind, in front, left, right, in, on, under, etc., always refer to our body itself or to objects that we perceive in analogy to our body. That is, the lived body does more than passively perceive; it actively resonates with the shape and position of objects.

Let us move on to the organization of our experience through *bodily action*. Moving our body in space is the basic form of acting; it therefore also provides the basis for categorizing other processes that we observe in the environment. This refers primarily to verbs that express actions such as moving, pushing, rising, falling; they denote forms of *operative intentionality* of our body (Merleau-Ponty, 1962, p. 372, 382), which we transfer to objects as if we were in their place. Thus we say that the fountain “springs into the air”, the house “nestles in the valley”, the path “twists” or “rises” etc. This also refers to bodily positions such as standing, lying, or resting, which we project onto perceived things. We say that the book is “lying” on the table, but the vase “stands” on the table because we perceive it as upright, assimilating it to our own upright position.

To extend the latter example: if we hear a simple sentence like “the book lies on the table”, its meaning arises for us through a combination of several components:

a) the evocation of two objects in our imagination, which includes not only their visual shapes but also their affordances for potential action, e.g., as something to grasp, open, and read (the book), something to sit at or put things on (the table), and so on;
b) the operative (motor, postural) intentionality of our body, which lets us implicitly grasp the state of “lying on”, namely as an experience of being “stretched out flat”, “supported by the ground”;
c) a syntactic structure that links a subject and a predicate in the way we experience ourselves as being or acting (“the book lies”, “the tree stands”, “the dog bites”, etc., as we would do ourselves).

So what we understand implicitly when we hear the sentence has the following meaning: the “thing to grasp, open, and read” lies “as I would” on the “thing to sit-at”. The sentence thus connects affordance-based terms with patterns of acting or relating, or in other words, *the syntax precisely imitates the operative intentionality of our body*.^2^

Of course, this is even more true for the complete subject-predicate-object structure of transitive sentences: “Peter—throws—the ball; the dog—bites—the bone; the ball—hits—the target”. In its basic grammatical structure, a sentence expresses a subject acting on an object in a way that we could on principle perform ourselves, or that transfers our action structure to inanimate objects. Through this very structure the sentence not only signifies, but enacts its own meaning and thus enables embodied understanding. Of course, verbs do not always express bodily activities or states, but the etymology often reveals the action origin. “I read the book” is not a sensorimotor action, but the corresponding German word *lesen* (to read) derives from *auflesen, sammeln* (to pick up, to collect), just as the Greek *légein* means “to pick up, to collect, to read”. So one reads by “picking up” the letters and words one after the other and thus “collecting” the meaningful sentences.

As we can see, language refers back to original contexts of action and transfers them into a new context, that of the symbolic use of signs, which still evoke their bodily origin. However, we must keep in mind that the linguistic articulation also dissolves the original situational unity of experience to a certain degree. The distinction between “I” and “ball” as subject and object is a secondary one, which is created by language and gradually solidified, for example, when the “I”, which at first actually only designates the acting subject or the speaker, is substantiated and hypostatized to an “I” or an “Ego”, as in Descartes' *res cogitans*. Nouns in particular tend to evoke substances, not processes or relations. This obscures the lived contexts from which they originally spring:

Even the word 'table', which seems to designate so clearly an object [...], is originally the signification of a relation, of a practical living intercourse, from which we only subsequently isolate the object 'table' by learning to deny the practical connection between the object of action and the person acting. ( Lorenzer 2002, p. 186; own trans.)

Opposing this hypostatization of concepts, pragmatism, phenomenology and ecological psychology in particular have rediscovered their original action context. Heidegger's concept of *Zuhandenheit* (being ready-to-hand), for example, restores things to their original embedding in practical contexts, as does Gibson's concept of *affordance*, which I have already mentioned. Both approaches allow us to understand the concepts of language in their original, relational, and embedded meaning.

## 4 Neurobiological confirmation

If we take a brief look at the emergence of symbolic language in human evolution and its neurobiology, it fits well with the concept of embodied cognition. A central principle of cultural development is *exaptation*, namely the use of existing biological resources for new functions. Thus, our conceptual system makes use of the sensorimotor system, which evolved long before the development of linguistic concepts and is now used for higher-level cognitive processes too (Deacon, 1998). This can be demonstrated in the human brain: More recent areas and centers employ older parts for new functions. For example, Broca's area, which is responsible for speech motor skills and articulation, has evolved from area F5, which coordinates visuomotor actions such as grasping in monkeys (Rizzolatti and Arbib, 1998; Pulvermüller and Fadiga, 2010). Thus, centers for sensorimotor coordination were increasingly used for linguistic concept formation during the evolution of Homo sapiens (Fitch, 2010; Kiverstein, 2020). The crucial connection between the sensorimotor system and language consists in *action simulation*: Sights or sounds that were regularly associated with an object triggered the activation of action plans for that object, i.e., an imaginary or simulated action (Gallese and Lakoff, 2005). Verbal sounds, as symbolic signs, then made it possible to separate the simulation from the presence of the object.

It is therefore not surprising that language processing in the brain has been increasingly shown to be functionally connected to sensorimotor systems. Thus, when thinking, speaking, or listening to words, the same sensorimotor areas in the brain are activated as for the practical engagement with the word-related objects (Gallese, 2008; Pulvermüller, 2005; Jirak et al., 2010). Thus, listening to sentences such as “the alarm sounded and John jumped out of bed” will activate areas both in the auditory and motor cortex related to alarms and jumping (Kaschak et al., 2006; Winter and Bergen, 2012). Let us look at some more examples:

- Listening to the words “grasp”, “shout”, “go”, or “kick” activates not only the receptive language areas, but also the motor centers that are involved in the corresponding actions (Buccino et al., 2005; Jirak et al., 2010). There is a definite somatotopy of language primarily in the pre-motor cortex: Pulvermüller (2005) identified differential fMRI-activity patterns here for consonant verbs that refer to the mouth, arms or legs like “lick”, “pick” and “kick”. In each case, the premotor cortex is differentially engaged in a topographical bodily pattern.
- When listening to verbs referring to hand movements (give, take, point, etc.) right-handed people show an activation of the left pre-motor cortex, left-handed people an activation of the right (Willems et al., 2010). This shows that the verbs are processed according to the actual bodily movement that one could perform. Moreover, it strongly suggests that they have already been learnt in this embodied way: “To give” meant originally “handing something over to mom with my right hand” (or left hand, in the other case).
- Glenberg et al. (2008) found that the *abstract usage* of verbs, as in the phrases “to give a reason', “to grasp a notion”, activates the motor system no less than the concrete usage. In other words, the latent bodily meaning continues to permeate the use of abstract terms, in this case verbs that are used metaphorically.

As we can see, there is strong neurobiological evidence for an enactive concept of language as being crucially based on bodily perception and action. In other words, language exploits the pre-existing multimodal character of the sensorimotor brain systems for simulating potential perception or action (Gallese and Lakoff, 2005).^3^

## 5 Higher-level concepts

Now one can argue that this bodily basis of concepts does not apply to higher levels of abstraction: there seems to be no embodied account of abstract terms like “truth”, “time”, “justice”, etc. However, we have already seen that the meaning of abstract terms can be based on bodily experience as well, namely on its metaphorical use. In their *conceptual metaphor theory* (CMT), Lakoff and Johnson proposed this not only for verbs, but also for nouns:

… even our most abiding concepts – time, events, causation, morality, and mind itself – are understood and reasoned about via multiple metaphors. In each case, one conceptual domain (say, time) is reasoned about, as well as talked about, in terms of the conceptual structure of another domain (say, space). (Lakoff and Johnson, 1980, p. 243)

The basic idea, then, is that the source domain of the respective metaphorization of concepts is embodied experience: for example, “time” is understood in terms of terms of bodily space and movement (forwards/backwards → future/past); the same applies to value concepts (up/down → good/bad), and so on. In what follows I will investigate some examples of embodied metaphors more closely, considering space, time, causality, and moral con-cepts. However, it should first be noted that Gibbs et al. (2004); (Gibbs 2006) already made important modifications to the theory: he argued that metaphors are not just fixed schemata, but embodied, context-sensitive processes that also integrate gesture and visual imagery. Thus, as he has also shown through psycholinguistic experiments, not every metaphor evokes the same mapping or embodied simulation (“good” is not always “up”, and vice versa). This corresponds to the fact that abstract concepts (space, worth, truth, etc.) refer to a more general context than concrete concepts (chair, stone, etc.), thus exhibiting greater variability and context dependency.^4^ Let us now look at some abstract concepts, before further discussing the possible limits of CMT.

### 5.1 The concept of space

As is well known, Kant considered space a pure, *a priori* form of intuition. However, it is difficult to imagine how we could arrive at a concept of space without embodied experience. This begins with the sensed voluminosity and movement of our body, for example, when we feel the broadening of the chest while breathing or the widening space between our arms when spreading them. Spatial experience continues in the touching of surfaces, where one's bodily sensing meets resistance and the groping conveys the spatial form of the object. Interoceptive, proprioceptive, kinesthetic, and exteroceptive sensations are integrated into a multimodal, bodily-sensorimotor space, which is simultaneously the space of self-awareness (Tsakiris, 2017; Ehrsson, 2020).

But even visual space only opens up through our own movement. In a classic experiment by Held and Hein (1963) on newborn (initially blind) cats, one group of kittens could move actively in the experimental environment; the others were only passively moved around in a small carriage. After a few weeks, the active kittens were able to move around normally, while the passive kittens were unable to orient themselves in space, stumbled around helplessly, and bumped into objects. This shows that only the moving—and at the same time sensing—organism forms the experienced space, namely from the coherently linked patterns of self-initiated motor activity and sensory feedback. The basic findings of Held and Hein have been repeatedly confirmed in animal and human studies: Movement is not merely an addition, but a necessary condition for the full development of spatial-visual abilities (Anderson et al., 2013; Oudgenoeg-Paz and Rivière, 2014; Smith et al., 2018).

Importantly, not only spatial perception, but also the acquisition of *space-related language* has been shown to depend on the development and degree of the infant's self-locomotion (Oudgenoeg-Paz and Rivière, 2014). The more infants engage in motor activity, the more they focus their attention on information needed to guide their movements. Accordingly, several studies have provided convincing support for a link between spatial cognition and spatial language (Landau and Hoffman, 2005; Wallentin et al., 2005). In his monograph “Concepts in the Brain” Kemmerer (2019) treats spatial concepts as a particular bridge between concrete, perceptually grounded categories (like “cup” or “chair”) and abstract domains (like “justice” or “freedom”). Spatial relations are *relatively abstract* because they are relational, context-dependent, and not directly perceivable as objects; yet they are still tightly connected to embodied sensorimotor systems. Accordingly, when people process linguistic expressions of spatial relations (e.g., “the cup is on the table”), neuroimaging studies show co-activation of linguistic and visuo-spatial regions (Kemmerer, 2019, p. 179ff). This suggests that spatial semantics employs embodied, modality-specific systems rather than an amodal symbolic store.

I have already mentioned the basic spatial orientation according to the body schema: right, left, forward, backward, up and down. Importantly, these orientations also imply an elementary attribution of meaning and value: for humans, attaining the upright posture not only means a central experience of self-control; it also means gaining overview and distance from things. ‘Upward' is therefore the positive direction of value, enhanced in joy or pride, diminished in sorrow or defeat. From this, Lakoff and Johnson (1980, p. 51) also derive the quantitative metaphor “more is up” and “less is down,” so that stock prices rise or fall, a company trends upwards or downwards, and so on. Similarly, “forward” means momentum, progress, success; “backward” represents weakness, regression, or defeat. This already points to the bodily basis of moral concepts (see below).

### 5.2 The concept of the container

There is a special form of spatiality, which is of crucial importance for the formation of categories and logical structures, namely the *container*. Containers convey the basic structure “inside – boundary – outside” and thus distinguish two spaces or sets of contained or non-contained objects. The concept is also based essentially on embodied experience. First, the body itself is a container whose incorporation or excretion, inhalation or exhalation is felt and perceived. Infants gain further experience of “inside” and “outside” with physical containers or boxes, which they constantly handle. These become the source domain for special metaphorical containers, namely *categories*. Categories are, in a sense, containers into which something is placed or from which something is removed (Lakoff and Johnson, 1999, p. 31). This is the basis of predication, namely the assignment of an object to a categorical “container”. When children learn to put red balls in one box and blue balls in another, they learn the operational scheme of an assignment that can be expressed as predication: “This ball is red”, “that ball is blue”. The categorization takes place as an embodied experience in various ways before linguistic predications can build upon it.

On this basis, a principle of Aristotelian logic can already be formulated, namely the law of excluded middle. For a ball can only be inside the box or outside it. More abstractly formulated: of two contradictory statements (p/not-p), one must be true—there is no third option in between. Similarly, *modus ponens* arises from the nesting of two containers: If all humans are mortal (A in B) and Socrates is a human (S in A), then Socrates is mortal ( → S also in B). We can see, therefore, that not only concepts and thus categories, but also logical operations with concepts have an embodied basis in the container scheme (*cf*. Lakoff and Johnson, 1999, p. 545).

The bodily basis of logical operations has already been emphasized by Piaget: According to him, children can only develop logical structures of thought because they have previously acquired corresponding *sensorimotor schemata* in embodied practice. This is because such schemata already contain transformations (e.g., turning, reversing, repeating) that later appear in thinking as logical operations (Piaget, 1948). Similarly, classification and sequencing are based on practical actions (e.g., collecting, arranging, stacking, sorting things) that are gradually transformed into mental operations through internalization (Piaget and Inhelder, 1959). To put it succinctly: *Logical operations are internalized and formalized bodily actions*.

Recently, Mondal (2022, 2024) has argued convincingly that the embodied and formal levels are not ontologically separate domains but different formats of the same underlying semantic content. Thus, the operation of *negation* can be derived from embodied experiences of “blocking”, “removing”, or “preventing” which map onto formal operators for negation. Similarly, the formal cognitive schemas for “possibility” or “future” are originally instantiated in embodied experience (possibility = “I can move freely”, future = “something not yet here but my body anticipates it”). Thus, embodiment supplies the content and constraints that are transformed into formal structures of logical thinking.

### 5.3 The concept of time

Let us move on to the concept of time, which, of course, cannot be separated from that of space and movement. Because the deliberate movement of the body, based above all on desire, constitutes a primal experiential unity of space and time, which can be expressed in the notion of “heading toward something”. It is the anticipation of the next future in the bodily protention that gives rise to a time differential, a “not-yet”, as it were. Bodily movement thus brings forth space as well as time; the body continuously “spatializes” and “temporalizes itself”; this is lived time.

It is therefore not surprising that Lakoff and Johnson see the conceptual metaphor for time in *bodily movement*. According to them, this metaphor has two opposing forms, namely (a) the movement of time itself, and (b) the movement of the subject (Lakoff and Johnson, 1999, p. 141, 145).

1. Let us take the first case: Time moves. The experiential basis for this is events which appear or come toward me from ahead. Therefore we speak in German of the *Zukunft*, or in French of “*l'avenir*”, i.e. literally that which ‘comes toward me' and ‘arrives at me'. We say: “The time will come”, but on the other hand time “flies by” or “passes”; the opportunity “has passed”. So the future lies ahead of me, whereas the space behind me is the space of the past.^5^
2. In the second case, we conversely see *ourselves as moving*, for example, when we say: “We are approaching Christmas,” “I have passed the deadline,” or: “Life is a river”. Here the river of time does not come toward us, but we move with it, or rather we ourselves are the river. The living movement of the subject itself is the passing of time. It is obvious that this second metaphoric of time is closer to the original experiential unity of space, time and movement, in which the body itself brings forth space-time.

In both cases, lived time thus becomes a quasi-spatial movement. On the other hand, bodily space, which is stretched out by experienced movement, can also be transformed into a geometric, static space. Lived time can then be plotted on a line that can be broken down into individual points and thus measured. However, this quantified time makes movement as a bodily anticipation of the future (in the sense of Husserl's protention) inconceivable.

Incidentally, this corresponds to a certain extent with Aristotle's conception, who in his Physics defines time as “the number of motion” —when time is spatialized, it can also be divided and measured in numbers. Of course, Aristotle lacks a sense of the foundation of time in bodily self-motion. This gives rise to Zeno's paradox of the flying arrow, which should actually be at rest, since it is located at a fixed point at every moment. Of course, the arrow moves, and we perceive this. But the metaphor of the spatial extension of time leads us astray as soon as we conceive of space as geometric rather than as the lived space of bodily movement.

One could now object, as Bergson (1889) did, that we have mistakenly spatialized time and thus missed the actual, indivisible time of duration (*durée*), the time that is lived. However, it is difficult to say how we should talk about time if not metaphorically—as the time that comes, passes, flies by, lasts, and can then also be measured. Ultimately, this metaphoric has its actual basis in the movement of our life itself, which brings forth a lived temporality; and this movement of life enables the movements of our body, which we then transfer to the movement of things and finally to the movement of time. The formation of the concept of time proves to be ambivalent: It originates from the spatiality of the moving body, but also leads to a reification that alienates lived time from the subject and establishes a “dominion of time,” namely of external, measurable or clock time (Theunissen, 1991; Fuchs, 2018).

The embodied basis of concepts of time has also been extensively studed by Kemmerer (2019, p. 217ff.). He notes that time is a highly abstract domain—unlike space, we cannot perceive time directly, only infer it through change and motion. Nevertheless, across languages, temporal meaning is systematically structured by spatial schemas (e.g. *before/after, ahead/behind, long/short time*). Kemmerer also extensively reviews neuroimaging research suggesting that the brain regions involved in spatial and motor processing are also recruited for temporal reasoning and language. Thus, when participants process temporal expressions (“Christmas is approaching”, “back in those days”), fMRI studies show co-activation of linguistic (left perisylvian) and parietal-motor regions—an instance of embodied simulation in abstract cognition. In short, temporal language largely reuses neural resources for space and action.

### 5.4 The concept of causality

We now proceed to the metaphoric of causality. We have already seen that we always experience and describe the movements and effects of things as *activities*, namely in analogy to our own bodily actions: The wind “pulls” on the branches, the water “drives” the mill wheel, one ball “pushes” another. If we wanted to describe such observations without using somatomorphic verbs, we would have to form complicated and difficult-to-understand phrases; on the other hand, the “push” of the ball is immediately understandable because we know from our own experience what a push is.

Our own bodily experience of exerting force, pressure, and movement also becomes the basis for perceiving something like *causality* in our environment.^6^ The proto-typical experience of causation is our experience of self-initiated manipulation of objects. It presupposes conscious agency as well as the experience of exerting force against external resistance and of the result of the action. Causality is originally *agent causality*.^7^ From this follows the metaphor: *causes are forces* (Lakoff and Johnson, 1999, p. 184)

This is projected onto objects (such as billiard balls colliding with each other), natural phenomena (the storm knocked the tree over), and then onto more abstract contexts (global warming is *causing* glaciers to melt). Natural phenomena thus become quasi-human agents, and the concepts of human action—pushing, pulling, lifting, giving, taking—become metaphors for our causal concepts in general. This applies to efficient causality, but it is easy to see that the Aristotelian concept of *final cause* also arises from a bodily metaphor: It follows from the goal directedness of our movements, namely to the place where they arrive and end, and this goal is transferred to the purpose of an external event. Finally, Aristotle's concept of *formal cause* is also derived from human activity, namely from the *creation* of a form or shape by the artist or the builder, hence in this case from embodied creativity.

The “interventionist theory of causation” fits into this context (Pearl, 2000; Woodward, 2003): According to this theory, we consider a factor X to be the cause of a change Y if an intervention that changes or varies X, while fixing all other influences, also changes Y (for example, when you turn the switch, the light comes on). Thus, it is about if-then relationships that are linked to an imagined or actual intervention or manipulation. Ultimately, the theory is based on an enactive conception: on the basis of our experiences of action, we place ourselves in the context in question, be it virtually or actually, and thus also endow the cause with our own agency.

### 5.5 Concepts of morality

Finally, I want to briefly discuss the embodiment of moral concepts, using the examples of justice and guilt. Etymology can again provide clues here. The noun “right” (in the sense of “having a right”) derives from the Indo-European roots *reg* (“to move in a straight line, to straighten up”) and *regtós* (“straight, upright”). It thus refers to a bodily operation, which implies an upright posture or gait and an experience of balance. This is transferred to the moral realm: to be a “righteous”, honest, or courageous person implies an inner or moral attitude embodied in a corresponding posture of standing or walking upright. Similarly, the more abstract meaning of “justice” or “equity” (German *Gerechtigkeit*) is based on the experience of bodily balance in the upright posture (as also represented in the scales of Justitia). Justice thus means an attitude that tries to give both sides what is due to them and to establish a balance.

The direction of an upright posture has further moral implications: it corresponds to higher values and “noble” sentiments, whereas reprehensible acts are committed out of “base motives.” The Platonic division of the soul's functions into the rational part (*logistikón*) in the head, the spirited part (*thymoeidés*) in the chest, and the appetitive part (*epithymetikón*) in the stomach also follows the value orientation along the vertical axis of the body.

Embodiment research can further substantiate the bodily basis of moral concepts, for example, through the connection between *guilt or moral failure* and *impurity*. Pilate washed his hands and thereby declared himself innocent of Jesus' death, and Lady Macbeth developed a washing compulsion after the murder of King Duncan. Recent research has shown that cleansing can actually wash away or alleviate feelings of guilt and mitigate moral (self-)condemnation (Zhong and Liljenquist, 2006; Lee and Schwarz, 2017; Lee et al., 2024). The central mediating emotion here is *disgust*, which is primarily directed at physical impurity, dirt, excrement, or decay, but which, in the course of cultural and moral development, increasingly takes on the function of moral revulsion. Cleansing rituals, atonement, and purification are closely linked in most religions. According to further studies, physical cleansing also weakens feelings of disgust in response to misconduct and thus mitigates moral judgment (Schnall et al., 2008; Tobia, 2015). The link between moral integrity and purity is as transculturally common as that between moral inferiority and impurity, if one only thinks of the *pariahs* or “untouchables” as the lowest class in India. “Pure” thus means “clean” as well as “innocent”, or to put in pointedly: “Morality is cleanliness” (Lakoff and Johnson, 1999, p. 307).

## 6 An enactive account of abstract concepts

I have now presented examples of the embodied experiential foundation of certain abstract concepts. However, Lakoff and Johnson's view that embodied metaphors are constitutive for the use of such concepts has not gone unchallenged. In an influential paper, Chatterjee (2010) critically reviewed neuropsychological and neuroimaging findings and concluded that the embodiment of concepts is actually partial, graded, and task-dependent. Thus, patients with motor cortex lesions can still comprehend action verbs and metaphors involving bodily movement, which means that conceptual access can survive motor impairment. Moreover, neuroimaging studies show that abstract concepts (e.g., justice, freedom, morality) correlate with inferior frontal and temporal activation, overlapping with linguistic and associative networks rather than motor or perceptual areas. This suggested a “weak embodiment” position: sensorimotor grounding can support but does not define conceptual representation. The processing of abstract concepts appears less embodied and more reliant on distributed, symbolic, multimodal or even amodal representations (Chatterjee, 2010).

A comprehensive meta-analysis of nearly 120 neuroimaging studies by (Binder et al. 2009, 2016) lead to a similar result. The authors concluded that while embodiment may underlie concrete concepts, abstract meanings rely more on linguistic, emotional, and social associations encoded in heteromodal neural systems. Overall, there is growing consensus that some kind of hybrid approach (modal/amodal, embodied/non-embodied, etc.) is needed; there could be gradations of embodiment, with formal and mathematical structures being least embodied (Dove, 2016; Mondal, 2022, 2024). (Kemmerer 2019) also argues for an intermediate position between strong embodiment and disembodied abstraction: spatial concepts, for example, are embodied in origin—they arise from the organization of sensorimotor systems that manage navigation, orientation, and manipulation. Through linguistic and cultural elaboration, however, they become partially abstracted, allowing humans to reason about “space” even without perceptual input.

Finally, a reconciliation of embodied and symbolic approaches was proposed by Borghi et al. (2018, 2025). They argue that abstract concepts are indeed linked to sensorimotor systems, but indirectly and through linguistic, affective, and social mediation. Words and concepts then function as “social tools” which extend primary embodiment: through dialogue and social interaction, words enable us to anchor meanings that go beyond direct bodily experience. Thus, Borghi et al. reformulate embodiment as multi-layered: (1) direct sensorimotor grounding for concrete concepts; (2) affective and interoceptive grounding for emotion-laden terms; and (3) social–linguistic grounding for highly abstract notions.

What are the consequences of this debate? The problems presented by conceptual metaphor theory suggest that a reformulation is indeed necessary. I propose that it can be based not only on an *embodied*, but also an *enactive* or *interactive* understanding of language. According to this view, the idea of concepts as fixed containers or schemata appears too static; rather, they should be seen as variable tools for navigating the landscapes of embodied and social interactions. The primary role of abstract words, then, would not be as signs corresponding to given schemata of metaphorical understanding. Rather, they are means of participating in the social world, which are derived from embodied experience in the way I have shown above, but extend into the realm of shared meanings, emotions, interactions, projects and expectations; in other words, they are means of *participatory sense-making* (Cuffari et al., 2015; Di Paolo et al., 2018).

Thus, like basic level concepts, abstract concepts are anchored in a situational context, albeit a more general one. Whereas the former focus on physically localized situations or objects, abstract concepts relate to more diffuse patterns and situations, especially emotional experience and social interactions or institutions (Barsalou and Wiemer-Hastings, 2005). Accordingly, abstract terms such as “peace,” “justice,” “democracy,” etc., extend far beyond their origins in sensorimotor or interoceptive experience. They are involved in an entire world orientation, namely an embodied attitude toward potential situations (e.g., in these cases, peacefulness, orderliness, political interest, etc,) as well as a certain atmosphere evoked by the field of associations and emotions connected to the respective term (e.g., calmness or relaxation, security, freedom, etc.). So-called “abstract” concepts are by no means neutral, detached or only intellectual. Rather, they always convey a bodily *felt sense* (Gendlin, 1998) of what it is like, for instance, to feel peace, to experience injustice, to hear about a murder, and so on. Accordingly, neurobiological studies have shown that abstract concepts entail affective processing to a significantly greater extent than concrete concepts (Kousta et al., 2011; Vigliocco et al., 2014; Mkrtychian et al., 2019).

Likewise, when we speak of “guilt”, we feel, at least subliminally, a bodily sense of burden, heaviness, wrongness, or impurity, but also of mutual obligation and responsibility, which suggests possible interactions such as reproach, remorse, or reconciliation. In other words, abstract terms such as “guilt,” “justice,” or “peace” correspond not only to certain physical and emotional experiences, but to specific forms of participatory sense-making or a *shared linguistic style* with which we inhabit the world as embodied beings (Cuffari et al., 2015; Irwin, 2017). Therefore, abstract thoughts about relationships, processes, possibilities, and counterfactual ideas do not imply direct simulation in the sense of a correspondence between words and sensorimotor programs; rather, they involve the use of concepts as *tools* or *skills* for engaging with a shared, enlanguaged world (Noë, 2016; Kiverstein and Rietveld, 2021).

To summarize: The embodiment of concepts should be understood as multi-layered, integrating interoceptive anchoring, sensorimotor simulation, emotional association, as well as participatory sense-making. While abstract concepts retain a basis in embodied experience that is transformed into conceptual metaphors, they transcend sensorimotor systems and encompass emotional and social references that connect them to a complex variety of intersubjective situations. In this way, they function as patterns available for enacting certain forms of sense-making; we need to understand them as embodied means and skills of participating in the shared lifeworld. There is thus a somatic-semantic continuum in which even abstract concepts can affect our experience, right down to the way we feel and breathe.

## 7 Conclusion

My aim was to show how embodiment and enactivism can contribute to solving the problem of “scaling up”, the transition from embodied experience to symbolic conceptual language, a transition that is both characteristic and constitutive of the human life form. Evolutionary evidence, neurobiological and embodiment research as well as phenomenological analysis of concept formation make it plausible that our linguistic concepts are constitutively based on bodily experiences and operations, so that even our most abstract concepts and verbs remain linked to this experiential context. Without their embodied basis, our concepts would indeed be empty, as Kant put it, for they would have no anchor point in our primary experience. As a free-floating, self-referential system of symbols they would be subject to the “symbol grounding problem”: The meaning of our language can only be based on the meanings that we experience as “embodied symbol users” (Jung, 2016) in our life contexts. This is also why the algorithmic synthesis of meaningful text, as performed through Large Language Models, always remains parasitic on the lived and participatory sense-making of humans which has accumulated in the data sources exploited by AI.

Human reason is crucially based on concepts, but it is not thereby disembodied, but rather constitutively embodied. And its concepts fit the world in which we live and act, because they have been formed on the basis of our sensorimotor and social interaction with it. Even higher forms of cognition are not based solely on an external assignment of purely symbolic propositions to the intended reality. Because the concepts which make up these propositions are not intelligible at all without our embodiment. What we consider to be true in a situation and therefore formulate in veridical propositions always depends on our embodied understanding of that situation. Epistemologically, this means that metaphysical realism, which would be based on a disembodied “view from nowhere,” must be rejected just as much as naive realism. What takes their place is an *interactive realism* of embodied subjects who communicate with each other about their interactions on the basis of bodily experience and metaphors (Fuchs, 2021).

This social, interactive basis of language cannot therefore be conceived without embodiment either. When children acquire language, words obtain their primary meaning through shared embodied practice, i.e., in situations of intercorporeality, joint attention, pointing gestures, and other ostensive cues (Tomasello, 1999; Fuchs, 2018). Even when verbal meanings can increasingly be detached from the concrete situation, all early language acquisition takes place against the backdrop of interactive situations: eating, washing, dressing, playing, building a tower, and so on. The child always learns first to engage in the practical situation at hand and to form common goals, and then classifies the language s/he hears in this context (Bruner, 1983; Nelson, 1996). The concepts s/he acquires in the process—water, food, tower, play, etc.—always contain a reference to shared practice. The same applies to the more abstract concepts that refer to general structures of this practice. Language ability does not develop from genetic predisposition, but must be—more than any other human ability—embedded in a communicative practice in order to flourish.

Words are the carriers of intersubjective meanings that have formed within a culture and differentiated into a complex reference system. However, meanings only exist *between* individuals, just as pointing one's finger only acquires its meaning through a shared gaze. Verbal communication is therefore not a transfer of symbolic meanings from one mind to another, but rather a “gesturing” or “pointing with words” in practical contexts. Not only embodied experience, but also the intercorporeal situation remains the basis of the meaning of concepts, whether practical or abstract—table and chair or time and justice.

## Data Availability

The original contributions presented in the study are included in the article/supplementary material, further inquiries can be directed to the corresponding author.

## Footnotes

1 This extension of the body schema to objects we interact with has also been confirmed in neurobiological studies (see Iriki et al., 1996; Carlson et al., 2010).

2 One might object that all these affordances and bodily conditions are far too complex to be present in the immediate understanding of the sentence. As we will see, however, there is now a lot of neurobiological evidence showing that this is indeed the case (see below).But apart from that, the question is how one could ever come to understand the meaning of *lying* at all, if not by “what I know from my own lying”, even if this embodied knowledge is only activated in the most remote way when hearing the word later on. For otherwise it would be very difficult and circuitous to explain what *lying* actually means, for example, “the spatial relation of an object being in close contact with another object underneath, touching it with its most extended side, whereas its smaller side remains free and upright.” And even then, we would run straight away into the symbol-grounding problem, for what the symbols “spatial”, “contact”, “touching”, “cover”, etc., in that definition mean could only be explained by even more complex definitions, and so on *ad infinitum*. Language cannot be a free-floating system of symbolic representations—it must ultimately be grounded in embodied experience.

3 It should be mentioned that Kemmerer (2019) has raised important methodological reservations about the generalizability of the neurobiological findings: many studies assume “common” categories that may not be universal. Kemmerer points to a large cross-linguistic variation in linguistic structures that calls this assumption into question: languages differ in how they encode body parts, agency, self-vs-others, motion of the body etc.

4 Further empirical applications of CMT to corpus linguistics have also been provided by (Steen 2010).

5 In some languages, such as those spoken in the Pacific region, it is the other way around: the past lies ahead of me, the future behind me. However, this does not change the spatial metaphorization of time.

6 In response to Hume's critique of causality, Schapp pointed to the phenomenal reality of causal relationships in perception: one sees how the elastic bowstring propels the arrow, or how the force of the arrow shatters the brittle windowpane. Just as one “... sees hard and soft, one also ‘sees' causality. [...] One sees more than the temporal sequence of events; one sees the connection between the events [...] it is physically present.” (Schapp, 1925, p. 46).

7 Etymologically, causality derives from the Latin *causa*, i.e., the word for the court trial: “Who did it?” is the primary question, and human actions and perpetrators are the original “causes”.
